# FoxH1 represses *miR-430* during early embryonic development of zebrafish via non-canonical regulation

**DOI:** 10.1186/s12915-019-0683-z

**Published:** 2019-07-30

**Authors:** Patrick Fischer, Hao Chen, Frederic Pacho, Dietmar Rieder, Robin A. Kimmel, Dirk Meyer

**Affiliations:** 1 Institute of Molecular Biology/CMBI, University of Innsbruck, Technikerstrasse 25, 6020 Innsbruck, Austria; 2 Division of Bioinformatics, Biocenter, Innsbruck Medical University, Innrain 80, 6020 Innsbruck, Austria

**Keywords:** Gastrulation, Nodal signaling, FoxH1, miR-430, MZT, Chromatin folding

## Abstract

**Background:**

FoxH1 is a forkhead transcription factor with conserved key functions in vertebrate mesoderm induction and left-right patterning downstream of the TGF-beta/Nodal signaling pathway. Binding of the forkhead domain (FHD) of FoxH1 to a highly conserved proximal sequence motif was shown to regulate target gene expression.

**Results:**

We identify the conserved microRNA-430 family (miR-430) as a novel target of FoxH1. miR-430 levels are increased in *foxH1* mutants, resulting in a reduced expression of transcripts that are targeted by miR-430 for degradation. To determine the underlying mechanism of miR-430 repression, we performed chromatin immunoprecipitation studies and overexpression experiments with mutant as well as constitutive active and repressive forms of FoxH1. Our studies reveal a molecular interaction of FoxH1 with *miR-430* loci independent of the FHD. Furthermore, we show that previously described mutant forms of FoxH1 that disrupt DNA binding or that lack the C-terminal Smad Interaction Domain (SID) dominantly interfere with miR-430 repression, but not with the regulation of previously described FoxH1 targets.

**Conclusions:**

We were able to identify the distinct roles of protein domains of FoxH1 in the regulation process of *miR-430*. We provide evidence that the indirect repression of *miR-430* loci depends on the connection to a distal repressive chromosome environment via a non-canonical mode. The widespread distribution of such non-canonical binding sites of FoxH1, found not only in our study, argues against a function restricted to regulating *miR-430* and for a more global role of FoxH1 in chromatin folding.

**Electronic supplementary material:**

The online version of this article (10.1186/s12915-019-0683-z) contains supplementary material, which is available to authorized users.

## Background

Gastrulation and hence formation of the three germ layers endoderm, mesoderm, and ectoderm are a key step in development from single-cell to multicellular organism. Among other pathways, dose-dependent signaling by the TGF-beta factor Nodal is central for germ layer induction and patterning. Throughout the vertebrate phylogeny, the loss of Nodal signaling leads to loss of endodermal and mesodermal cell fate [1–3]. A central step in Nodal signaling is the ligand-induced phosphorylation and subsequent nuclear translocation of Smad2. Within the nucleus, Smad2 forms a complex with Smad4 and interacts with different transcription factors, each targeting the SMADs to a different set of target genes ([4–6], reviewed in [7]). The transcription factor FoxH1 was the first SMAD-interacting protein to be identified. Genetic studies established conserved requirements for FoxH1 in mediating Nodal activities related to mesoderm induction and left-right patterning [8–12]. Consistent with the genetic requirements, molecular studies identified several mesoderm-related transcription factors (e.g., tbxta, tbx6, foxA3, pitx2) and signaling molecules (e.g., FGF8, FGF3, Wnt11) as conserved FoxH1 targets. FoxH1 is further required for modulating Nodal signaling intensity, range, and duration by directly regulating Nodal and Lefty encoding genes, with the latter being a Nodal antagonist [8, 13–16].

FoxH1 binds chromatin via the conserved canonical (CAN) consensus motif AATMCACA. CAN binding sites critical for mediating Nodal signals are further characterized by close-by SMAD binding sites (SBS) [8, 16–19]. Currently, it is not entirely clear whether FoxH1 can bind chromatin without the Smad2/3 interaction. Recent work in pluripotent mouse P19 cells showed that binding of FoxH1 to SBS-associated CAN enhancers is strictly Smad2 dependent [20]. However, studies in human embryonic stem cells and very recent findings in frog embryos showed that FoxH1 can be pre-positioned at specific enhancers and that the subsequent interaction with activated Smad2 can induce a release or a switch from repression to activation of associated genes [9, 21].

The FoxH1 protein has two functionally well-defined domains, an N-terminal forkhead domain (FHD) for DNA-binding and a C-terminal domain required for interaction with activated SMADs (SID, Smad interaction domain). An additional medially positioned conserved EH1 motif was recently shown to mediate Nodal-independent transcriptional repression via interaction with Groucho/TLE factors [22, 23]. Genetic analyses in zebrafish revealed slightly different phenotypes in *foxH1* mutant embryos lacking either a functional FHD (*sur*/*schmalspur*) or the SID (*mid*/*midway*). In maternal and zygotic (MZ) mutant embryos, both types of *foxH1* mutations cause defects in axial mesoderm formation, a loss of ventral neural fates, ventral body curvature, and synophthalmia. However, while axial mesoderm is partially disrupted in MZ*sur* (= *foxH1*^*m786*^) mutants [10, 24], it is missing in MZ*mid* (= *foxH1*^*Pr1*^) mutants and the embryos display additional defects in somite patterning. Since MZ*mid* mutants can be rescued to an MZ*sur* like phenotype by injection of *sur* mutant *foxH1*^*m786*^ mRNA, it was suggested that DNA-binding defects in *sur* mutants might be partially compensated by the interaction of the FoxH1^*m786*^/SMAD complex with other DNA-binding proteins [11]. Consistent with this option, canonical FoxH1 binding sites are frequently found in association with binding sites for other Smad2-interacting transcription factors such as Eomes and Mixl1 [12].

Interestingly, previous ChIP analyses revealed that the majority of FoxH1 peaks in fact lack CAN FoxH1 consensus motifs [12, 25]. The molecular nature and functional relevance of these peaks, termed non-canonical (NC) peaks, have not been addressed so far. In this study, we identified the *miR-430* loci as targets for NC interaction with FoxH1 and we show that this interaction is relevant for FoxH1 dependent *miR-430* repression.

In zebrafish, 415 different miRNAs in 44 families have been found so far ([26], overview in [27]). Among those, three isoforms of *miR-430* could be distinguished (a, b, c), which differ in their central and terminal nucleotide sequence, but are homologous in the 3′ region and target recognition site at the 5′ end ([28–30], overview in [27]). The majority of *miR-430s* are transcribed from a large cluster on chromosome 4 that contains more than 50 copies of *miR-430* [31]. Like most vertebrate miRNAs, *miR-430* are also transcribed as long primary transcripts (*pri-miR-430*). These transcripts undergo a maturation process: cleavage into hairpin-structured precursor miRNA (*pre-miR-430*) by a protein complex of Drosha and its co-factor DGCR8, transport into cytoplasm, generation of a 22-nucleotide long imperfect RNA duplex by Dicer and co-factors, and loading into the RISC (RNA-induced silencing complex) ([31]; overview in [27]). miR-430 family members are highly expressed at the onset of gastrulation (5 h post fertilization, 5hpf) and stay expressed during gastrulation and somitogenesis [32–36]. The early embryonic expression of *miR-430* is required for clearance of maternal RNAs at the time when zygotic expression starts (midblastula transition; Additional file 1: Figure S1 [30, 37]). To this end, *miR-430* targets hundreds of transcripts for deadenylation and degradation [34]. Furthermore, *miR-430* is involved in fine tuning and regulation of Nodal signaling, namely by targeting mRNA encoding the Nodal agonist *nodal-related 1* (*ndr1*; *squint)* and the antagonist *lefty2* [36, 38, 39].

Here we show that NC-binding of FoxH1 at *miR-430* loci correlates with a downregulated expression of all three *miR-430* subtypes and with a downregulation of *miR-430*-targets in the MZ*sur* mutant. We further show that the FHD and the SID-containing C-terminus of FoxH1 are both required for *miR-430* repression and that mutant forms lacking function of one of these domains dominantly interfere with this activity. Our data demonstrate a novel physiologically relevant requirement for FoxH1 in miR-430 regulation, and they provide a first insight into the mechanism underlying non-canonical gene regulation by FoxH1. The results imply a hypothetical model of indirect FoxH1 activity on *miR-430* loci that includes chromatin folding effects.

## Results

### ChIP-seq and microarray revealed non-canonical FoxH1 regulation of *miR-430*

**Fig. 1:**
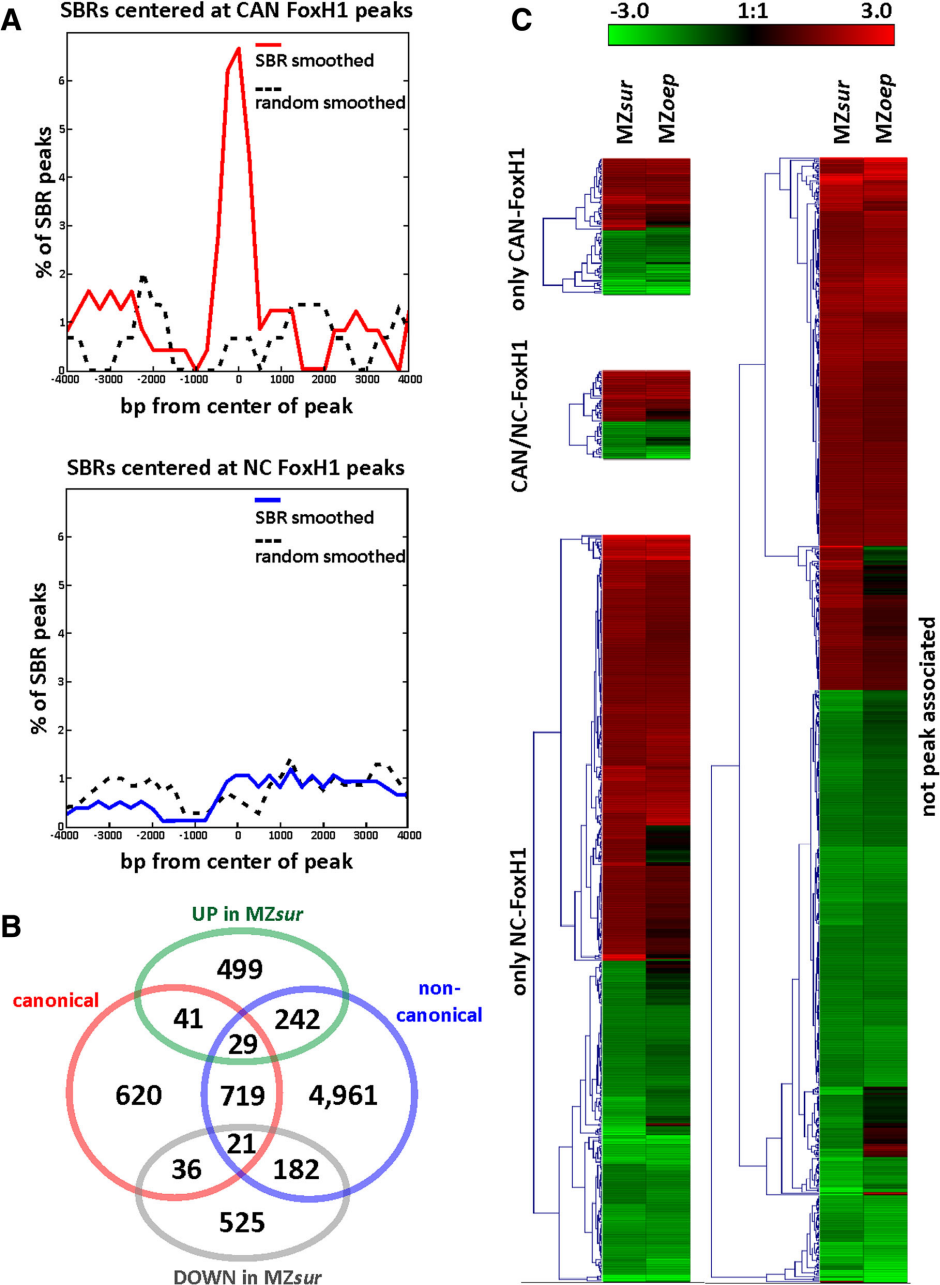
FoxH1 is associated with canonical (CAN) and non-canonical (NC) target sites. **a** FoxH1 CAN-peaks but not NC-peaks co-localize with SBRs. **b** Indication of the numbers of genes with more than twofold up (UP) or down (DOWN) regulation in MZ*sur* mutants associated with CAN, NC, or CAN+NC peaks of FoxH1. **c** Heatmap comparison of all FoxH1 peak-associated (left panels) and not FoxH1 associated (right panel) genes with more than twofold changed expression in MZ*sur* (left-side) as compared to wild-type embryos. Note that most genes show very similar changes of expression in MZ*oep* mutants (see right-side for comparison)

To gain insight into the function of FoxH1 during early embryonic development, we performed a combination of expression and ChIP-seq analysis on 6hpf epiboly-stage zebrafish embryos. The ChIP analyses [40] revealed 8,342,137 high-quality reads that could be mapped to more than 16,000 peaks (*p* < e^−4^) in the zebrafish genome (Zv9/danRer7). De novo motif prediction algorithms from two different toolsets (MEME-ChIP and RSAT) [41–43] confirmed the presence of the well-established FoxH1 consensus motif in about 14% (2421; Additional file 2: FoxH1-peaks and Annotation-50 k + 20 k) of these peaks [8, 16–19]. Correlation analysis of FoxH1 peaks with published Smad2 binding regions (SBRs) [44] further confirmed overlap between SBRs and FoxH1 peaks containing the consensus motif (termed CAN-peaks) but not with peaks lacking the motif (termed NC-peaks) (Fig. 1a; Additional file 3: SBR to FoxH1 peaks).

To correlate chromatin binding with requirements for FoxH1-dependent gene regulation, we performed microarray-based transcriptome analyses*.* Comparison of expression data from 6hpf epiboly-stage wild-type (biological duplicates) embryos with that of MZ*sur* mutants (biological triplicates) revealed a total of 1575 genes with at least twofold changes in expression (Fig. 1b; Additional file 4: Microarray vs. ChIP).

Consistent with the expected primary role of FoxH1 in Nodal signaling, we also find that the majority of these genes (76%) show a similarly changed expression in Nodal signaling-deficient MZ*oep* mutants (Fig. 1c; Additional file 4: Microarray vs. ChIP). To identify potentially direct FoxH1 targets, all regulated genes were analyzed for the presence of FoxH1 peaks within 50 kb upstream and 20 kb downstream of the transcriptional start site (TSS). FoxH1 peaks were found for 551 of the genes with twofold changed regulation in MZ*sur* mutants (Fig. 1b; Additional file 4: Microarray vs. ChIP). From those, 127 genes were associated with Smad2/FoxH1 co-binding, which included most of the previously reported Nodal/FoxH1-activated genes (*ndr1*, *lft2*, *pitx2, flh*, *foxa3*, and *lhx1a*) [3]. Surprisingly, the majority of FoxH1-regulated genes associated with NC-peaks. As FoxH1 has been mainly associated with gene-activating functions, we expected direct targets to be mainly downregulated in the mutants. However, similar numbers of down- and upregulated peak-associated genes in MZ*sur* and MZ*oep* mutants suggested that CAN- and NC-FoxH1 peaks both contributed to Nodal/FoxH1-dependent gene activation and repression (Fig. 1c).

**Fig. 2:**
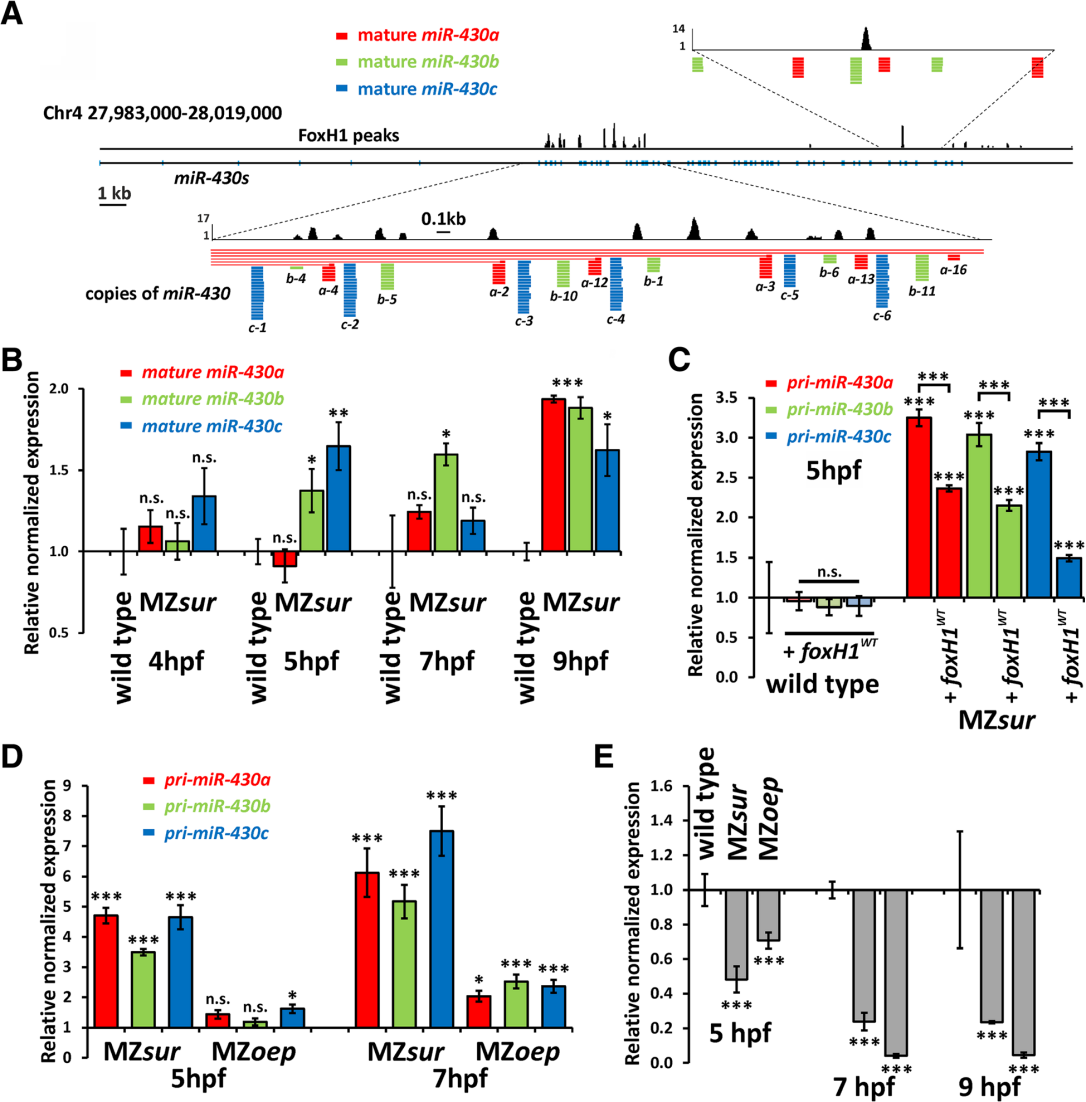
FoxH1 regulates *miR-430* expression in a negative manner. **a** Genomic organization of the *miR-430*-containing region on chromosome 4. FoxH1 peaks localize to the *miR-430a*, *b*, and *c* repeat cluster. Enlargements indicate positions of transcripts for *miR-430a* (red), *miR-430b* (green) and *miR-430c* (blue) which were directly extracted from the UCSC browser [45]. **b** Temporal changes of mature *miR-430* levels in MZ*sur* embryos between 4hpf and 9hpf as measured by RT-qPCR relative to wild-type embryos. Starting at 5hpf, levels of mature *miR-430* are increased in the mutant. **c** The upregulation of *pri-miR-430* forms in MZ*sur* mutants can be partly rescued by injection of *foxH1*^*WT*^ mRNA. Addition of FoxH1 in wild types does not influence the expression level. **d** All three *pri-miR-430* isoforms are upregulated in MZ*sur* mutants at the indicated time points. Loss of Nodal signaling in the *oep* mutant does not significantly change expression levels. All data normalized to respective wild type control. **e** Control RT-qPCR shows for MZ*sur* and MZ*oep* mutants a highly significant downregulation of *gsc* expression as formerly shown [11, 46]. All qPCR data were calculated from biological triplicates except for **b** (2 biological replicates). Error bars indicate standard error (SEM). Bio-Rad CFX Manager 3.1 software was used to calculate relative normalized expression, standard error, and significance (n. s. *p* ≥ 0.05; *p < 0.05; **p < 0.01; ***p < 0.001). For individual values, see also Additional file 5: Individual qPCR values

Among NC-peaks, we noted several prominent peaks within the *miR-430* “a, b, c” repeat cluster (Additional file 2: FoxH1-peaks and Annotation-50 k + 20 k; Fig. 2a [45]). To determine potential requirements of FoxH1 in *miR-430* regulation, we analyzed expression of mature *miR-430* isoforms (a, b, c) by using a poly-A tailing approach [47]. Consistent with a general role of FoxH1 in *miR-430* repression, all three *miR-430* isoforms were significantly increased in gastrula stage MZ*sur* mutant embryos (Fig. 2b; Additional file 5: Individual qPCR values). Increased *miR-430* transcription in MZ*sur* as compared to wild type was further confirmed by direct RT-qPCR analyses of selected subsets of *pri-miR-430* transcripts for the three isoforms (Fig. 2c; Additional file 5: Individual qPCR values).

To confirm that the repressive effect is specific to FoxH1, we further tested whether injection of *foxH1*^*WT*^ mRNA into MZ*sur* embryos would lower *pri-miR-430* levels. Consistent with a specific function, the injected *foxH1*^*WT*^ mRNA reduced *pri-miR-430* levels (Fig. 2c; Additional file 5: Individual qPCR values).

Next, we asked whether *miR-430* regulation by FoxH1 is Nodal signaling-dependent. Consistent with a role of Nodal signaling in *miR-430* repression 7hpf MZ*oep* mutants (Fig. 2d; Additional file 5: Individual qPCR values) show increased *pri-miR-430* transcript levels. However, this increase was less prominent as compared to MZ*sur* mutants. As a control, we verified that the canonical Nodal target *goosecoid* (*gsc)* is reduced in MZ*oep* and MZ*sur* mutants (Fig. 2e; Additional file 5: Individual qPCR values). Together, these data reveal a novel requirement for FoxH1 in *pri-miR-430* repression, and they suggest that the *sur* mutation has Nodal signaling-independent effects on *miR-430* regulation.

### Mutations in the FHD and SID of FoxH1 dominantly interfere with *miR-430* regulation

**Fig. 3:**
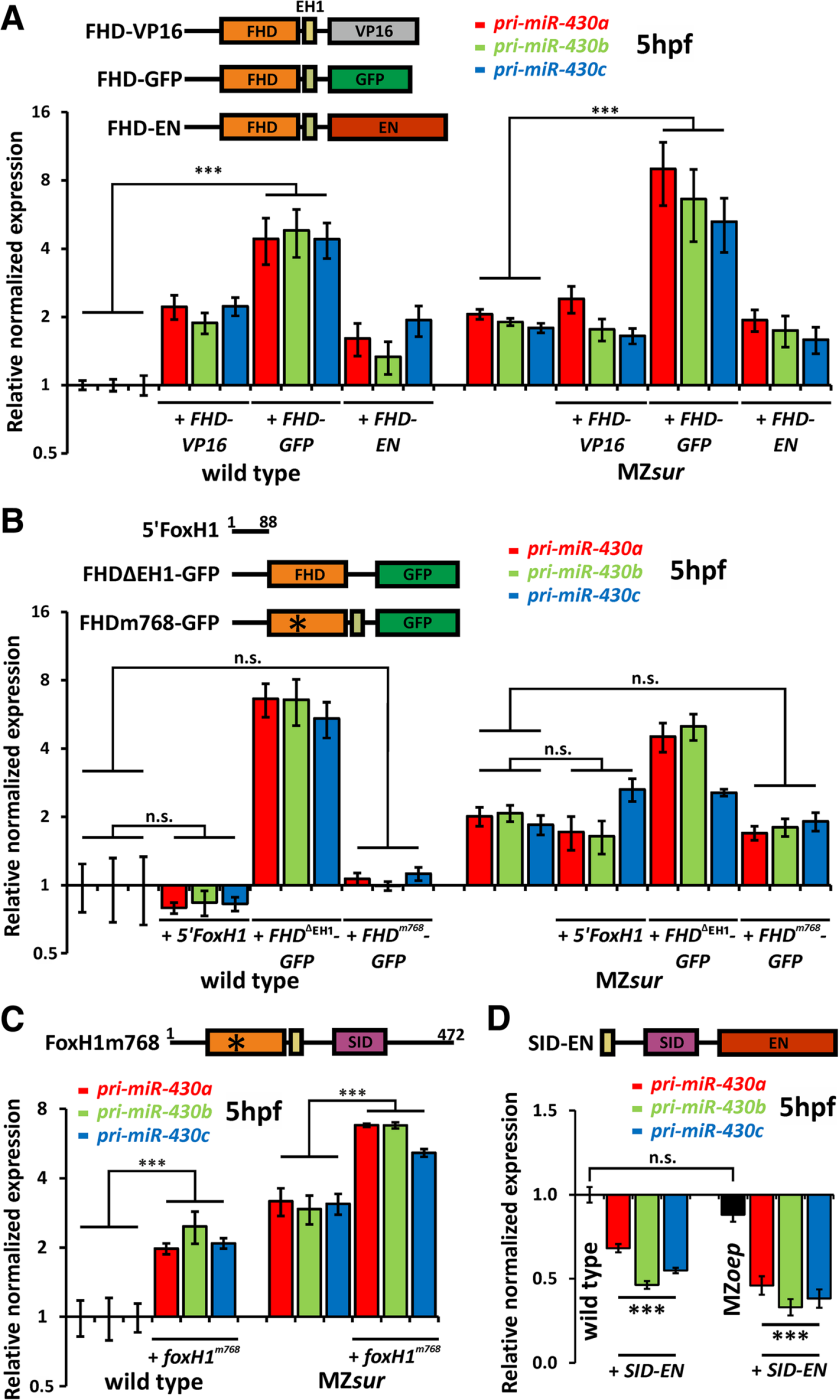
Mutated FoxH1 proteins for SID or FHD reveal dominant-negative effects on pri-miR-430 at 5hpf. **a** The FHD fused to VP16, EN, or GFP leads to enhanced upregulation in wild-type and MZ*sur* mutants. Note highest upregulation for *FHD-GFP* constructs. **b** Sequences 5′ and 3′ to the FHD do not influence expression levels. A s*ur*-mutated form of FHD fused to GFP does also not interfere with the expression level. **c** Addition of mutant proteins without DNA binding ability enhances the upregulation in wild type and MZ*sur* mutant embryos. **d** Injection of *SID-EN* in both wild type and MZ*oep* mutant embryos resulted in the downregulation of *pri-miR-430* expression. The full-length protein consists of 472 aa. FHD (orange) is wild type or mutated to *sur* allele (*). SID (purple) can be wild type or replaced by VP16 (gray), GFP (green), or EN (brown). Olive-colored box following the FHD represents the EH1 domain which was included in earlier VP16, GFP, and EN constructs [10]. Error bars indicate standard error (SEM). All experiments were performed as biological triplicates of 5hpf embryos injected with the indicated mRNA at 1–2-cell stage. Relative normalized expression, standard error, and significance were calculated with the Bio-Rad CFX Manager 3.1 software (n. s. *p* ≥ 0.05; ***p < 0.001). For individual values, see also Additional file 5: Individual qPCR values

The lack of canonical binding motifs within the *miR-430* associated peaks suggests that FoxH1 is not directly interacting with these sites. To further test this, we performed RNA injections with FHD-containing constructs (Additional file 6: Overview of FoxH1 constructs) previously shown to cause robust activation (FHD-VP16) or repression (FHD-EN) on CAN targets (Fig. 3a; Additional file 5: Individual qPCR values) [10, 11]. Analysis of selected CAN-FoxH1 targets in injected embryos showed the expected up- and downregulation by injections of *FHD-VP16* and *FHD-EN*, respectively, and virtually no effect following injection of a *FHD-GFP* control construct (Additional file 7: Figure S2). In contrast, and consistent with an indirect regulatory effect, all three types of injections (*FHD-VP16*, *FHD-EN* and *FHD-GFP*) caused increased *pri-miR-430* levels (Fig. 3a; Additional file 5: Individual qPCR values) with *FHD-GFP* showing the strongest activity not only in wild type but also in MZ*sur* mutants*.*

The data suggest that the common FHD-containing N-terminus of FoxH1 in these constructs interferes with *pri-miR-430* repression in a dominant negative manner. To clarify which parts of the proteins mediate the dominant negative activities, we tested additional GFP fusion constructs (Fig. 3b; Additional file 5: Individual qPCR values, see also Additional file 6: Overview of FoxH1 constructs). In particular, we addressed functions of the conserved C-terminal region that includes the EH1 motif and of the non-conserved N-terminal part. We found that the 88 most N-terminal amino acids of FoxH1 (5′-Foxh1) were not sufficient to influence *pri-miR-430* levels and that the removal of the EH1 motif (*FHD*^ΔEH1^*-GFP*) did not prevent interference with *pri-miR-430* repression. These data suggest that the FHD, while not directly interacting with the *miR-430* loci, is critical for *miR-430* regulation. To further test if this critical activity depends on the DNA-binding properties of the FHD, we injected a *sur*^*768*^ mutant variant of FHD-GFP (termed FHD^m768^-GFP; Additional file 6: Overview of FoxH1 constructs). The single amino acid exchange in FoxH1^m768^ has previously been shown to specifically prevent binding to FoxH1-consensus motifs [10]. Since FHD^m768^-GFP had no major effect on the regulation of *pri-miR-430* (Fig. 3b; Additional file 5: Individual qPCR values), this suggests that CAN-DNA interaction is important for the dominant negative function of FHD-GFP.

The data imply that SID-depleted fusion proteins with a wild-type FHD bind to an undefined CAN binding site and thereby prevent repressive interactions of this CAN-site with the *miR-430* loci. This further suggests that the SID-containing C-terminus of FoxH1 might be required for mediating contact with the *miR-430* loci. In this case, overexpression of the FoxH1 ^*m768*^ protein, with an intact SID but defective FHD, should also interfere with *pri-miR-430* repression by occupying the *miR-430* loci-specific contact site. Consistent with this notion, injection of *foxH1*^*m768*^ mRNA caused twofold increased expression levels of *pri-miR-430* in wild type and surprisingly also in MZ*sur* embryos (Fig. 3c; Additional file 5: Individual qPCR values). To directly address whether a FHD-depleted FoxH1 protein is able to interact with the *miR-430* loci, we tested a fusion construct between the C-terminal part of FoxH1 and the EN-repressor domain (termed *SID-EN*; Additional file 6: Overview of FoxH1 constructs). In contrast to *foxH1*^*m768*^ that caused increased *pri-miR-430* levels, the injection of *SID-EN* resulted in strongly reduced *pri-miR-430* levels (Fig. 3d; Additional file 5: Individual qPCR values). Since SID-EN lacks known DNA-binding motifs, we reasoned that its interaction with the *miR-430 loci* might be mediated through interaction with SMAD2/3 proteins. However, downregulation of *pri-miR-430* by *SID-EN* was also seen in MZ*oep* mutant embryos (Fig. 3d; Additional file 5: Individual qPCR values) which lack phosphorylated and thereby nuclear SMAD proteins. These results suggest a SMAD-independent interaction of the SID with the *miR-430* loci.

Together, these data suggest that FHD and the SID-containing C-terminus of FoxH1 have unique functions in mediating repressive contact between distal chromatin structures and that injection of FoxH1 proteins lacking one of these domains interfere with this activity in a dominant negative manner.

### Injection of *FHD-GFP* increased severity of MZ*sur* mutant phenotype

**Fig. 4:**
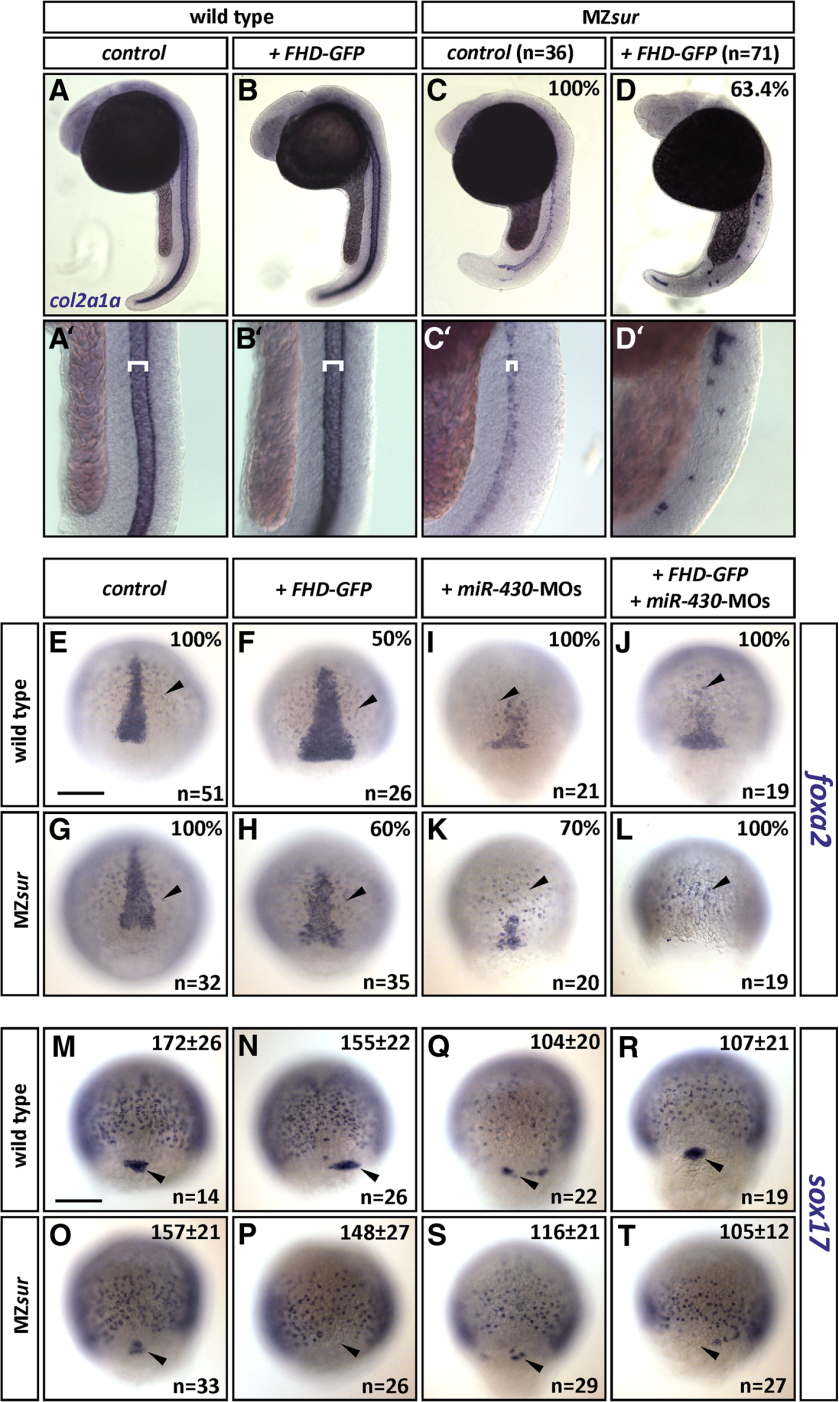
FHD-GFP interferes with severity of the MZ*sur* mutant phenotype. **a**–**d** Wild-type (**a**, **b**) and MZ*sur* mutant (**c**, **d**) embryos at 24hpf. *col2a1a* in-situ staining in wild-type (un-injected control (**a**) or injected with *FHD-GFP* (**b**)) shows wild-type notochord of expected width (white brackets in enlarged sections **a**′ and **b**′). In uninjected MZ*sur* embryos the width is reduced (**c**′). Injection of *FHD-GFP* in MZ*sur* mutants enhances the phenotype (**d**/**d**′; note reduced size and additionally discontinuity of staining). **e**–**l** *foxa2* in-situ hybridizations show reduction of axial mesoderm formation in MZ*sur* mutants. Injection of *FHD-GFP* causes a broadened axial signal in 50% of wild type embryos, but no reduction of axial cells (**f**), and strengthens the effect in 60% of the MZ*sur* mutants (**h**). *dre-miR-430* morpholinos (MOs) massively reduce axial *foxa2* signals in both genotypes (**l**, **k**). Co-injection of *FHD-GFP* and MOs also results in a decreased staining (**j**, **l**) when compared to *FHD*-*GFP* injection (**f**, **h**). Percentage of embryos showing the same phenotype as in the image is given (upper right). **m**–**t** *sox17* in-situ hybridizations show only slight reduction of endoderm after injection of MOs (**q**–**t**). Number of forerunner cells (black arrow) is reduced in MZ*sur* control and after MOs injections (**o**, **q**, **s**). *FHD-GFP* lead to complete loss of forerunner cells (black arrow) in the majority of MZ*sur* embryos (***p***, **t**; see also Additional file 8: Statistical analysis of forerunner cells). Numbers of *sox17*-positive cells seen in dorsal view and standard deviation are given (upper right) as well as number of analyzed embryos (*n*) (lower right). Size bars: 200 μm. *γ* value was changed to 0.8 in each picture

In previous studies, the injection of *foxH1*^*m768*^ mRNA into MZ*mid* mutants was found to convert the more severe MZ*mid* phenotype into the weaker MZ*sur* phenotype. It was therefore suggested that FoxH1^*pr1*^ might be inactive while FoxH1^m768^ retains residual FoxH1 activities via the intact SID [11]. Our studies show that both types of mutations interfere with *miR-430* regulation. Since the loss of the SID in FHD-GFP, resembling FoxH1^*pr1*^, has stronger effects on *miR-430* regulation as compared to FoxH1^m768^, we reasoned that the stronger phenotype of MZ*mid* could be associated with the stronger dominant-negative effects of the SID-truncated protein. Consistent with this notion, we found that injection of *FHD-GFP* into MZ*sur* mutants strongly increased the expressivity of the mutant phenotype. WISH analyses for the axial marker *col2a1a* in 24hpf embryos confirmed a major effect of *FHD-GFP* on notochord formation in MZ*sur* mutants (Fig. 4a–d). MZ*sur* mutants lack the floor plate and hypochord and show some variable notochord defects in the tail and anterior trunk [11]. Still, these embryos showed nearly continuous *col2a1a* staining in the trunk notochord, while more than 60% of *FHD-GFP* injected MZ*sur* embryos displayed only patchy *col2a1a* signals. Analyses of *foxa2* expression in gastrula stage embryos indicated that *FHD-GFP* prevents formation of early axial mesoderm in MZ*sur* (Fig. 4e–h). We found that the number of *foxa2-*positive axial cells was strongly reduced in *FHD-GFP* injected MZ*sur* as compared to control and un-injected MZ*sur* embryos. Notably, *FHD-GFP* also caused broadened axial signal in about 50% of the injected control embryos, possibly indicating defects in gastrulation movement. The *foxa2* stains also suggested that *FHD-GFP* has mild effects on endoderm formation (arrowheads in Fig. 4e–h). Further analyses of the endoderm marker *sox17* confirmed a slightly reduced amount of endoderm cells, and in addition, they revealed strongly reduced number of forerunner cells in *FHD-GFP* injected embryos (Fig. 4m–t; Additional file 8: Statistical analysis of forerunner cells).

As the severity of *foxH1* mutant phenotype appears to correlate with the level of *miR-430* upregulation, we next tested whether an injection of *miR-430* blocking *dre-miR-430* morpholinos (MOs) [36] is able to attenuate the phenotype of MZ*sur* and *FHD-GFP* injected MZ*sur* (Fig. 4i–l). However, the morpholino injection had the opposite effect and caused strongly reduced and even absent axial *foxa2* signals in MZ*sur* and *FHD-GFP* injected MZ*sur*, respectively (Fig. 4k, l).

### FoxH1 regulation of *miR-430* prevents maternal clearance

**Fig. 5:**
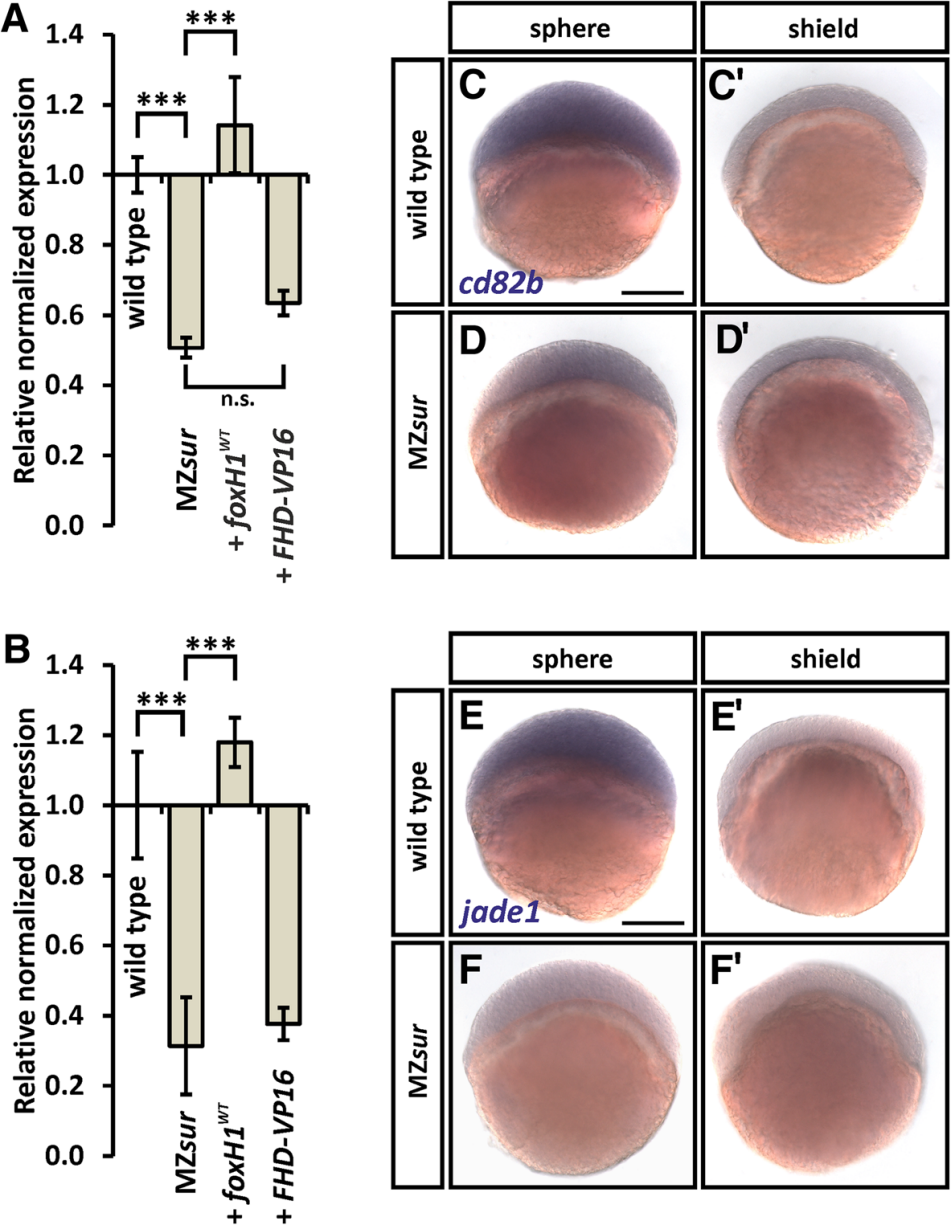
FoxH1 blocks maternal clearance of *cd82b* and *jade1.* **a**, **b** RT-qPCR analysis of *cd82b* (**a**) or *jade1* (**b**) in embryos at 50% epiboly with indicated genetic background. Massive reduction of expression is shown in MZs*u*r mutants which cannot be rescued by injection of *FHD*-*VP16* mRNA but is rescued via injection of *foxH1*^*WT*^ mRNA. Error bars indicate standard error (SEM) from 2 biological replicates. Calculation of relative normalized expression, standard error, and significance was made with the Bio-Rad CFX Manager 3.1 software (***p < 0.001; n.s. *p* ≥ 0.05). For individual values, see also Additional file 5: Individual qPCR values. **c**–**f** WISH for *cd82b* and *jade1* in wild type embryos (**c**, **e**) shows staining at sphere stage, but not at shield stage when miR-430 becomes active (**c**′, **e**′). In MZ*sur* mutants (**d**, **f**), weak or no staining is visible for all stages indicating the negative role of FoxH1 in regulating miR-430 activity at early embryonic stages. Size bars 200 μm

Finally, we asked whether regulation of *miR-430* by FoxH1 is functionally relevant in the developing embryo. Based on previous studies, we expected that the increased *miR-430* levels in MZ*sur* mutants or RNA-injected embryos would have effects on maternal transcript clearance [34, 47]. To determine functions of FoxH1 in maternal transcript clearance, we analyzed expression of two maternally deposited miR-430 targets, namely *cd82b* [48, 49] and *jade1* [50, 51]. Expression analyses of 4–5hpf embryos by RT-qPCR (Fig. 5a, b; Additional file 5: Individual qPCR values) and WISH (Fig. 5c–f) show that transcripts levels of *cd82b* and *jade1* are significantly lower in MZ*sur* mutants as compared to wild-type embryos and that this phenotype is reduced by injection of *foxH1*^*WT*^ mRNA. To exclude a possible direct regulation of these genes by FoxH1 or by canonical FoxH1 targets, we also analyzed expression in *FHD-VP16* injected embryos. The similar *cd82b* and *jade1* levels in un-injected and *FHD-VP16* injected MZ*sur* embryos emphasize the indirect connection between FoxH1 and *cd82b/jade1* and are consistent with the important intermediary role of *pri-mir-430* upregulation, as seen following injection of *FHD-VP16* (Figs. 3a and 5a, b). In summary, this shows that FoxH1-dependent repression of *miR-430* is biologically relevant for the regulation of miR-430 target genes during maternal clearance.

## Discussion

Here we describe a novel role for the transcription factor FoxH1 in negative regulation of *miR-430*, and we provide evidence for a regulatory mechanism that is different from the previously described canonical role of FoxH1 in TGF-beta/Nodal signaling.

More than 20 years ago, FoxH1 was identified as the first Smad2-interacting transcription factor mediating transcriptional gene activation downstream of TGF-beta/Nodal signaling. Since then, FoxH1 has become established as a central transcriptional regulator of Nodal-induced mesendoderm induction and left-right patterning. Furthermore, a broad panel of genes were identified that are directly regulated by FoxH1/Smad2 via binding to proximal CAN binding sites [8–19]. Only recently, genome-wide ChiP analyses provided a more complex picture of highly dynamic FoxH1 chromatin occupancy in the early embryo. While these studies confirmed the importance of Nodal/Activin-induced CAN-interactions, they also revealed a much larger number of possibly Nodal/Activin independent NC-DNA interactions of unknown function [12, 25]. Our analyses now provide first evidence for a functional relevance for NC-interactions in gene regulation. Among other NC-targets, we identified the *miR-430* cluster as a novel in vivo target that is repressed by NC-FoxH1 interaction. Consistent with a Nodal independent function, we find that MZ*sur* mutants display a stronger upregulation of *miR-430* as compared to Nodal signaling-deficient MZ*oep* mutants (Fig. 2). Functional relevance for *pri-miR-430* repression by FoxH1 was confirmed by showing that the increased *pri-miR-430* levels in MZ*sur* mutants correlate with a reduction of maternal transcripts that are targeted by miR-430 for de-adenylation and degradation. Our data suggest that the ubiquitous distribution of FoxH1 in the early embryo is required to restrict the level of miR-430 induction at the onset of zygotic gene expression. The patterned zygotic expression of FoxH1 during gastrulation and early somitogenesis [10] further implies that FoxH1 in addition to its role in mediating Nodal signaling might function in spatio-temporal controlled attenuation of miR-430-dependent transcriptional silencing or mRNA clearance. Importantly, miR-430 is dampening not only maternal, but also a large number of zygotically expressed transcripts with various functions in early embryogenesis. Interestingly, this includes *lft2* and *ndr1*, which are both also directly regulated by CAN-FoxH1/Smad2-mediated gene activation [36, 38, 39]. Since both mechanisms for *lft2* and *ndr1* regulation, CAN-FoxH1-dependent feedback-activation and miR-430-mediated transcript decay, are critical for balancing Nodal signaling, our data hint for a complex multilayered role of FoxH1 in directly and indirectly controlling and mediating Nodal activities in the early embryo. For future studies, it will be important to further characterize direct and indirect activities of FoxH1 on Nodal signaling and to study compensatory interactions between these activities.

### MZ*sur* and MZ*mid* embryo phenotypes may not reflect complete loss of FoxH1

Our data suggest that the currently available *foxH1* mutants *sur* and *mid* both can interfere with gene regulation and that some aspects of the MZ*sur* and MZ*mid* mutant phenotypes might not be seen in a truly null allele. In a previous study, it was shown that the stronger MZ*mid* phenotype is rescued to an MZ*sur*-like phenotype by an injection of *foxH1*^*m768*^ but not by *foxH1*^*mid*^ RNA. Since injection of *foxH1*^*mid*^ RNA in wild-type embryos caused no obvious defects, it was suggested that FoxH1^m768^ retains residual activity that is missing in FoxH1^*mid*^ [11]. Our results confirmed a generally normal morphology of wild-type embryos injected with the *foxH1*^*mid*^-like *FHD-GFP* mRNA, while they also revealed a broadened notochord in these embryos during gastrulation. Most relevant, we find that injection of *FHD-GFP* converted MZ*sur* embryos into more severe MZ*mid-*like embryos (Fig. 4). This notion is also consistent with the observed increase of *miR-430* levels by *FHD-GFP* not only in wild type but also in MZ*sur* mutants. Therefore, our data hint for a more complex mechanism in which FoxH1^*m768*^ may prevent or attenuate the stronger dominant-negative or possible neomorphic effects of FoxH1^*mid*^. Interestingly, we also noticed that *FHD-GFP* has a stronger effect on *miR-430* regulation as compared to *FHD-VP16* and *FHD-EN* (Fig. 3a)*.* Since the corresponding *sur*-mutant *FHD*^*m768*^*-GFP* is inactive, we consider *FHD-GFP* activities as specific to the FHD. Possibly, the robust activation and repression of CAN-FoxH1 targets by *FHD-VP16* and *FHD-EN,* respectively, is able to compensate for aspects seen in *FHD-GFP* injections.

While we focused on *miR-430* regulation to determine functions of the different FoxH1 domains, our combined ChIP and expression data hint for a much broader role for NC-FoxH1 interactions in gene regulation. Consistent with this notion, we propose that the changed *miR-430* levels are not the major cause for the phenotypic differences between MZ*sur* and MZ*mid*. In case of a primary cause, the morpholino knock-down of *miR-430* should have reduced mesoderm defects in *FHD-GFP* injected MZ*sur*. Instead, a strong reduction or complete loss of axial mesoderm was observed in these embryos (Fig. 4i–l). Since *miR-430s* target hundreds of mRNAs, the morpholino injections might cause a dominant phenotype that overrides the expected axial mesoderm rescue by *miR-430* reduction. However, in wild-type embryos, injection of *dre-miR-430* MOs was shown to reduce Nodal signaling by causing premature and increased translation of Lefty proteins ([36], see also Fig. 4l). While the complete loss of axial mesoderm in *FHD-GFP* and morpholino co-injected MZ*sur* is consistent with a reduction of Nodal signaling by Lefty proteins, the strongly reduced *lft1/2* mRNAs levels in MZ*sur* mutants argue against such a mechanism [36]. In this context, the large number of NC-FoxH1 peaks suggest that FHD-GFP, similar to its effect on *miR-430* expression, might interfere with other regulators of axial mesoderm formation. Consistent with this notion, our data suggest NC-interaction with Wnt, FGF, and retinoic acid signaling components (Fig. 4; Additional file 2: FoxH1-peaks and Annotation-50 k + 20 k).

In conclusion, these studies show that for a detailed understanding of the early molecular events underlying mesendoderm formation, it is important to have a true null allele for FoxH1. In corresponding mutants, overexpression of distinct FoxH1 variants could be used to separate CAN- and NC-FoxH1 functions and to determine the molecular factors responsible for the phenotypic differences between MZ*sur* and MZ*mid* [10, 11, 24].

### A new hypothetical model for FoxH1 regulation of *miR-430* locus

The data generated in this study suggest that *miR-430* repression by FoxH1 requires interaction of FoxH1 with at least two distinct genomic loci (Figs. 1 and 3). They also suggest that two major protein domains of FoxH1, the FHD and the SID, have specific functions in mediating these interactions and that this activity is independent of the central EH1 domain, which was shown to mediate Nodal-independent gene-repression via direct interaction with Groucho/TLE co-repressors (Fig. 3) [22, 23]. As SID-EN is able to repress the *miR-430* cluster independent of Nodal-signaling (demonstrated in MZ*oep* mutants; Fig. 3d), our data suggest a SID interaction with chromatin that is independent of activated SMAD2.

**Fig. 6:**
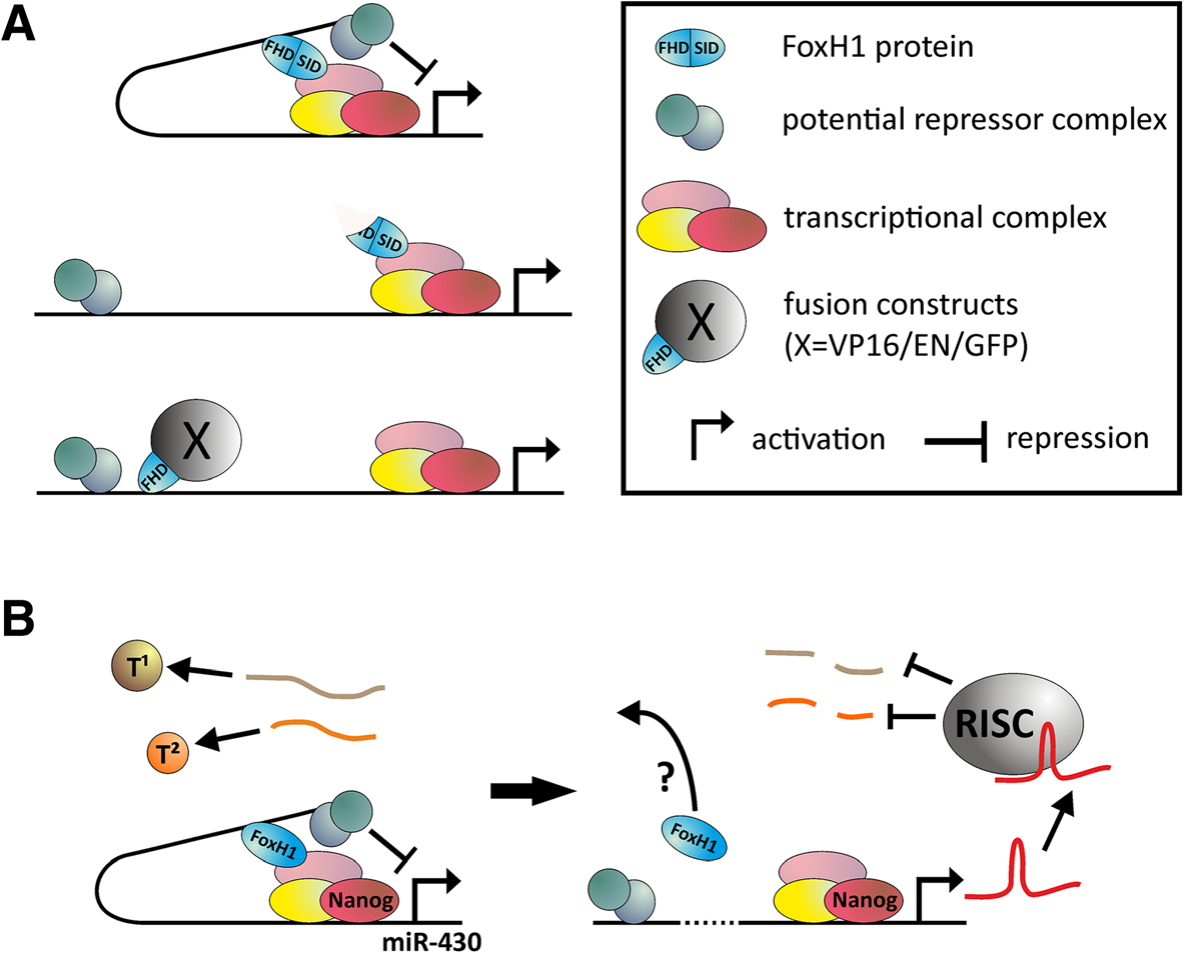
A proposed model for the role of FoxH1 in the regulation of miR-430 activity. **a** Wild-type FoxH1 causes chromatin looping, preventing expression of *miR-430*. In MZ*sur* mutants lacking the FHD, this loop is absent because FoxH1 binds only to the *miR-430* cluster. If FoxH1 lacking a functional SID or FHD (due to mutation (m786) or replacement (VP16/GFP/EN)) is injected into wild types, higher expression of *miR-430* occurs due to loss of looping and inhibitory regulation. **b** FoxH1 occupies non-canonical (NC) target sequences at *miR-430* loci to induce chromatin scaffolding and prevent *pri-miR-430 expression*. With the onset of gastrulation, FoxH1 leaves the site, allowing miR-430 to become active and repress its targets (T^1^/T^2^)

Therefore, we propose a novel role of FoxH1 in connecting the *miR-430* cluster with a distal regulatory element which then mediates repression per se, or another protein or protein complex does the work, as shown in our hypothetical model (Fig. 6a). Accordingly, we further propose that the currently available mutants dominantly interfere with chromatin scaffolding by association with contact sites either at the distal CAN-motif (Mid) or at the *miR-430* cluster (Sur). At the onset of MZT, the inhibitory effect of FoxH1 is overcome by an unknown mechanism. Since Nanog, Pou5f1, and SoxB1 are known positive regulators of *miR-430* [47], FoxH1 and the changed chromatin scaffolding may prevent binding of these factors to *miR-430* enhancer sites or block translation at these sites. It is not known whether Nanog/Pou5f1/SoxB1 bind at *miR-430* loci from the earliest developmental stages or can only occupy these binding sites once the FoxH1-initiated change in chromatin scaffolding disappears.

In the last decade, it has become clear that NC-DNA interactions of transcription factors are often associated with chromatin looping, bringing distantly located DNA domains closer together ([52], reviewed in [53–56]). Therefore, regulatory elements can influence distal genes not only on the same chromosome but also on different chromosomes [57]. Various examples in model organisms such as *Drosophila* and mice, as well as human cell culture systems, demonstrate high plasticity in the formation of the so-called Extremely Long-Range Promotor-Promotor Interactions (ELRIs), which are associated with the initiation and/or maintenance of gene activity, even during the earliest developmental stages ([57–62], reviewed in [63]). While the data we present are well explained by an involvement of FoxH1 in chromatin folding, further experiments are necessary to confirm this role. Several methods have been described for studying chromatin loops: 3C and derivatives, ChIA-PET, DNA fluorescent in situ hybridization (overview in [64–66]), and CRISPR/Cas9 guided in vivo chromatin labeling [67, 68]. By using one or a combination of these methods, potential FoxH1-associated loops can be identified. Due to the complexity of the *miR-430* loci, identifying relevant chromatin loops might not be as straightforward as for single and well-defined CAN-sites of a given transcriptional factor. However, our data also suggest that NC-FoxH1 activities are not restricted to *miR-430.* Consistent with a possibly more global role of FoxH1 in early chromatin folding, we find that a large number NC-FoxH1 peaks are associated with genes that are either up- (242/6154 peaks) or downregulated (182/6154 peaks) in MZ*sur* (Fig. 1c). In preliminary ChIP-qPCR studies that were set up to confirm selected NC-interactions of FoxH1, we noticed that enrichment of NC-peaks was variable in 4 and 5.5hpf samples while CAN-peaks were similarly enriched in these samples. In this context, the recently reported high number of dynamic FoxH1 chromatin interactions in early *Xenopus* development provides a hint for a possible conserved mechanism regulating dynamic interaction of FoxH1 at NC-sites [22, 23].

## Conclusions

Overall, our data show that FoxH1, in addition to its established function downstream of TGF-beta/Nodal-signals [8–12], can regulate gene expression via indirect SID-mediated chromatin interaction. By identifying the microRNA-430 family members as functionally relevant targets for non-canonical FoxH1 regulation, our data place FoxH1 upstream of miR-430-regulated processes such as maternal clearance and balancing of Nodal signaling. In addition, the widespread distribution of NC binding sites of FoxH1, found not only in our study, but also in a recent ChIP analysis performed in *Xenopus* [22], hint for a more global role of FoxH1 in early embryonic chromatin folding. Therefore, this new knowledge of NC-FoxH1 functions provides us with important new insights into the molecular and epigenetic mechanisms underlying early zygotic gene regulation.

## Methods

### Zebrafish lines

The following lines were used: MZ*sur*^*m768/m768*^ [69], MZ*oep*^*m134/m134*^ [70], and Tü (Tübingen) as wild-type control. Embryos were raised at 28.5 °C and staged to hours post-fertilization or percentage of epiboly as described [71].

### mRNA injections

*foxH1*^*WT*^, *eGFP*-*foxH1*, *FHD-VP16*, *FHD-GFP*, *FHD-EN* (all described in [10]), *FHD*^ΔEH1^*-GFP*, *foxH1*^*m768*^, *foxH1*^*m768*^*-GFP*, *5’foxH1*, and *SID*-*EN* were synthesized with the SP6 mMESSAGEmMACHINE in vitro transcription kit following the manufacturer’s protocol (Life Technologies, AM1340). Embryos were injected at 1–2-cell stage with 2 nl (= 90 pg) of each mRNA.

### Morpholino injections

Morpholinos against *dre-miR-430a*, *b*, and *c* were a kind gift from Caroline S. Hill [36]. Stock solutions of 2 mM were diluted for injection at a 1:12 ratio in water. Two nanoliters of a mixture of the three MOs was injected in embryos at the 1–2-cell stage.

### Chromatin immunoprecipitation assay (ChIP)

We used MZ*sur*^*m768/m768*^ embryos for the assay to exclude occupation of FoxH1 binding sites by endogenous FoxH1 proteins.

ChIP experiments were performed as described [72, 73] with minor changes. In total, 6000 *foxH1* mutant embryos (MZ*sur*^*m768/m768*^) were injected each at 1–2-cell stage with 8 ng/μl (~ 30 pg/embryo) of *eGFP-foxH1* mRNA, encoding full-length FoxH1 with an N-terminal eGFP tag. Embryos were collected and cross-linked at 6hpf. Chromatin was extracted and sheared into 100–200 bp fragments with the Bioruptor Sonicator (Diagenode, Belgium).

Anti-eGFP antibody (Torrey Pine Biolabs, 0715119, Protein A purified rabbit IgG) was pre-blocked and added (10 μg/1000 embryos) to the cross-linked samples and incubated overnight at 4 °C. The next day, Dynabeads (Life Technologies, 10007D) were added to IP down protein-DNA complexes. Validated ChIP-DNA samples were amplified following the protocol of LinDa [74, 75]. The concentration and fragment size of final amplification products were analyzed on a Bioanalyzer High Sensitivity DNA chip.

### High-throughput sequencing and data analysis

LinDa-amplified ChIP-DNAs were sequenced on an Illumina HiSeq2000 at BGI (https://www.bgi.com/global/sequencing-services/epigenetics/chip-seq/) and produced a total of 24,932,016 raw clean 50 bp single-end reads. These reads were then processed and filtered to remove low-quality reads and artifacts (e.g., residual LinDa adapters) resulting in 12,430,636 high-quality reads which were mapped to the zebrafish genome assembly Zv9/danRer7 using the BWA (v0.6.2) short read mapper [76].

### Peak calling

Read alignments were first filtered using samtools to only retain uniquely mapping reads by removing all reads that map to multiple positions with equal alignment score. Then, peaks were called using MACS 1.4.2 [77] setting the *p* values cutoff to 1e^−4^ and disallowing duplicate tags (reads) at the same position to avoid amplification biases.

This produced 23,724 raw peaks, of which 8446 had a *p* value <1e^−5^. These peaks were further filtered such that they did not overlap by more than 50% of their length with a non-UTR Ensembl exon and that they did not contain the following simple repeats: CTCTCTCTCTCTCTCTCTCTCTCTCTCT, AGAGAGAGA [GA] AGAGAGAGAGA, GAGAGAAA. This finally resulted in 16,908 peaks.

### FoxH1 motif de novo analysis

The FoxH1 consensus binding motif was obtained by using meme-chip from the MEME suite [41] and the RSAT suite [42]. Sequences from peak regions that did not overlap with repeat-masked regions or genomic regions annotated as exons in the Ensembl database [78] but overlapped with conserved regions in fish and were within 5.5 kb of a TSS, were used as input sequences for motif prediction.

### Gene expression microarray

Total RNA was extracted and purified from wild-type, MZ*sur* and MZ*oep* embryos using Qiagen RNeasy Mini Kit (74104). Three biological replicates were used for MZ*sur*/MZ*oep* and two for wild type, all of them showed RIN value > 8 when analyzed with Agilent 2100 Bioanalyser. Agilent’s Zebrafish Oligo Microarrays (V2), P/N G2519F (Agilent Microarray Design ID 019161) were hybridized according to the manufacturer’s protocol. Raw data were analyzed using the Agi4x44PreProcess-Package (“R” package v1.22.0.) and the LIMMA-Package [79, 80]. Background correction mode was “half”; for normalization, the “quantile” option was used. The “Mean Signal” from the AFE software was used as signal, the “BGMedian Signal” as background. Data quality was confirmed using MA plots (data not shown).

### FoxH1 target gene annotations

Genes were annotated as potential direct targets of FoxH1 by running our in-house developed peak annotation tool adapted from [81] and written in Perl. Briefly, the tool annotates ChIPseq peaks applying the following association rules: each RefSeq gene (Zv9/danRer7) was assigned a basal regulatory domain 5 kb upstream and 1 kb downstream from the TSS. This basal domain could also overlap with basal domains from other genes. The basal domains were then extended in both directions to the nearest non-overlapping basal domain but no more than 50 kb upstream and 20 kb downstream from the TSS. Peaks within such regulatory domains (basal and extended) were associated with the corresponding genes. Genes associated with FoxH1 motif-containing peaks were defined as the “canonical FoxH1 regulated” genes.

### Analysis of FoxH1-SMADs direct targets

The Smad2 SBRs were obtained from [44]. The original data based on Zv7/danRer5 was transposed to Zv9/danRer7. The transposed SBRs were then re-annotated and associated with genes. Association rules described above (− 5 kb – TSS – + 1 kb + extension − 50 kb – TSS –+ 20 kb) were applied. These SBRs were then centered at CAN-FoxH1 peaks, and their distribution was plotted in 250 bp bins at the given distance to FoxH1 peaks. For building the random profiles, we generated the same number of random peaks as real peaks and analyzed them correspondingly.

### Genome-scale identification of potential FoxH1/SMAD targets

The sequences around CAN-Foxh1 peaks were scanned with known SMAD consensus binding motifs derived from Genomatix matbase (www.genomatix.de) and SMADs/FoxH1 pairs in which a SMAD motif (V$Smad3.02, V$Smad.01, V$Smad3.01, and V$Smad4.01) was located no more than 100 bp 5-prime of the FoxH1 motif were extracted and annotated.

### Mature miRNA extraction and RT-qPCR

The total RNA was extracted and purified by miRNeasy Kit (Qiagen, 217184) from 30 embryos. cDNA synthesis and qPCR were performed following a previously published protocol [82]. The oligo 5′-GCAGGTCCAGTTTTTTTTTTTTTTTCTACCCC-3′ was used for cDNA synthesis by Superscript III kit (Life Technologies, 18080-051). The miR-103 was used for sample normalization in mature microRNA RT-qPCR. Primer data can be found in Additional file 9: qPCR primer.

### RNA isolation and cDNA synthesis for pri-miRNA

TRIzol (Life Technologies, 15596026) was used to prepare total RNAs following the manufacturer’s protocol. After DNA digestion by RNase-free DNaseI, total RNA was cleaned up by EtOH purification. One microgram RNA was used as template for reverse transcription using Maxima First Strand cDNA Synthesis Kit for RT-qPCR (Thermo Scientific, # K1641).

### Quantitative real-time PCR for pri-miRNA

For each probe, 10 μl of 1:10 diluted cDNA was mixed with 4 μl HOT FIREPol Eva Green Mix (Solis Biodyne, 08-31-00001), 5.5 μl water, and 0.5 μl of one of the primer mixes in Additional file 9: qPCR primer (100 pmol/μl). Following protocol was used: initial step, 95 °C for 15 min; 40 cycles: 95 °C 15 s, 60 °C 30s, 72 °C 20 s.

The data analysis is described elsewhere [83, 84]. If not indicated, differentially wild-type control was used for normalization. Significance was calculated using the program Bio-Rad CFX Manager 3.1 (Bio-Rad Laboratories, USA) (n. s. *p* ≥ 0.05; *p < 0.05; **p < 0.01; ***p < 0.001). Microsoft Excel and CoralDraw were used for graphic composition.

### Whole mount in situ hybridization

For whole-mount in-situ hybridization, *cd82b-*, *jade1*-, *col2a1a*-, *foxa2*-, and *sox17*-mRNA were used for generating digoxigenin-UTP (Roche Applied Science, 11175025910) labeled antisense probe. Staining followed a recently described protocol [85] using NBT/BCIP as dye substrate. For reducing variability, all wild-type or MZ*sur* mutant embryos at different stages were stained in the same tube; probe and staining solutions were made for all and distributed to the single tubes.

For photo documentation, embryos were cleared in glycerol and imaged using a Leica CTR6000 microscope equipped with a Leica DFC 300FX camera (Leica Microsystems GmbH; Wetzlar, Germany). CoralDraw was used for assembling figures. Single dorsal view images were used for quantification of *sox17*-positive cells.

## Additional files

Additional file 1: **Figure S1.** *miR-430* expression starts at midblastula transition. (PDF 76 kb)

Additional file 2: FoxH1-peaks and Annotation-50k+20k. (XLSX 1476 kb)

Additional file 3: SBR to FoxH1 peaks. (XLSX 76 kb)

Additional file 4: Microarray vs. ChIP. (XLSX 778 kb)

Additional file 5: Individual qPCR values. (XLSX 90 kb)

Additional file 6: Overview of FoxH1 constructs. (PDF 241 kb)

Additional file 7: **Figure S2.** Regulation of CAN-target *pitx2* and *fgf3* by different constructs. (PDF 303 kb)

Additional file 8: Statistical analysis of forerunner cells. (XLSX 10 kb)

Additional file 9: qPCR primer. (XLSX 9 kb)

## Data Availability

All data generated or analyzed during this study are included in this published article, its additional files, and publicly available repositories. Following additional files are included: Additional file 1. pdf: Figure S1. *miR-430* expression starts at midblastula transition. Additional file 2. xlsx: FoxH1-peaks and Annotation-50k+20k Additional file 3. xlsx: SBR to FoxH1 peaks Additional file 4. xlsx: Microarray vs. ChIP Additional file 5. xlsx: Individual qPCR values Additional file 6. pdf: Overview of FoxH1 constructs Additional file 7. pdf: Figure S2**.** Regulation of CAN-target *pitx2* and *fgf3* by different constructs. Additional file 8. xlsx: Statistical analysis of forerunner cells Additional file 9. xlsx: qPCR primer Following datasets are available at public repository: ChIP data on Gene Expression Omnibus (GSE133990;[40])
