# Supplementary material for: FoxH1 represses miR-430 during early embryonic development of zebrafish via non-canonical regulation
Source: BMC Biol. 2019 Jul 30;17:61. doi: 10.1186/s12915-019-0683-z (PMC6664792; doi:10.1186/s12915-019-0683-z)
Supplement: Supplementary file 1 — Figure S1. miR-430 expression starts at midblastula transition. (PDF 76 kb) [file 12915_2019_683_MOESM1_ESM.pdf]

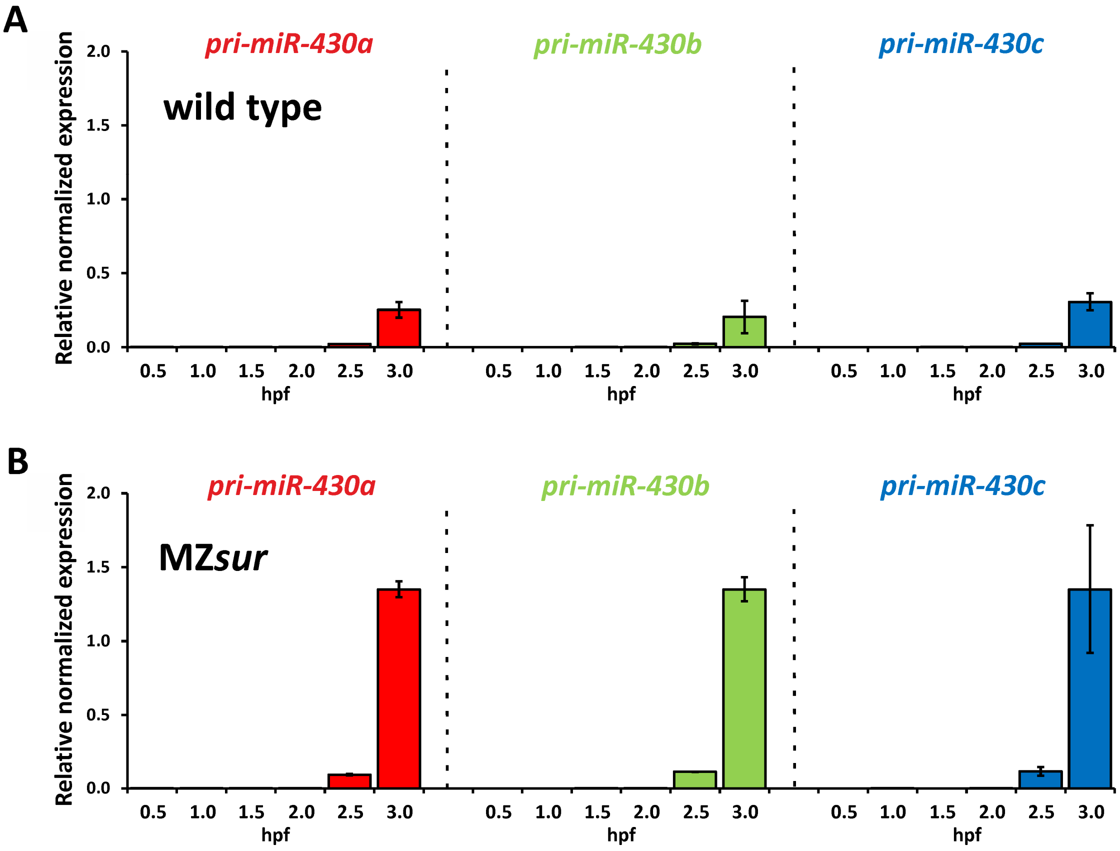

Fig. S1:

miR-430 expression starts at midblastula transition**.** Relative normalized expression of the *pri-miR-430* isoforms was measured at the indicated timepoints. The beginning of upregulation in wild type (*a*) and MZ*sur* mutants (*b*) correlates with the midblastula transition at about 3 hpf, validating previous studies [30, 37]. Error bars represent standard error (SEM) from 2 biological replicates. Individual values see also Additional file 5.xlsx: Individual qPCR values.
