# Supplementary material for: FoxH1 represses miR-430 during early embryonic development of zebrafish via non-canonical regulation
Source: BMC Biol. 2019 Jul 30;17:61. doi: 10.1186/s12915-019-0683-z (PMC6664792; doi:10.1186/s12915-019-0683-z)
Supplement: Supplementary file 6 — Overview of FoxH1 constructs. (PDF 241 kb) [file 12915_2019_683_MOESM6_ESM.pdf]

Name	Schematic Representation	Description
**FoxH1 wt**	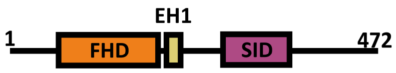	Wild typical FoxH1, full sequence, containing all domains [10]
**FHD-VP16**	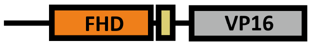	C-terminal fusion of the FHD and EH1 domains with activation domain VP16 [10]
**FHD-EN**	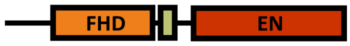	C-terminal fusion of the FHD and EH1 domains with repression domain Engrailed [10]
**FHD-GFP**	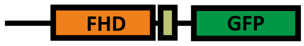	C-terminal fusion of the FHD and EH1 domains with GFP [10]
**5’FoxH1**	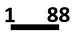	most 88 N-terminal amino acids before FHD domain [this work]
**FHDΔEH1-GFP**	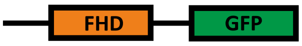	C-terminal fusion of the FHD domain with GFP (without EH1 domain) [this work]
**FHDm768-GFP**	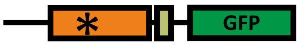	C-terminal fusion of the FHD and EH1 domains with GFP; FHD containing the *sur* mutation (R94→H94); unable to bind DNA via FHD [this work]
**m768**	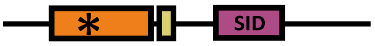	*sur* allel; SID and EH1 wild typical; FHD contains *sur* mutation(R94→H94); unable to bind DNA via FHD; intact SID and EH1 [this work]
**SID-EN**	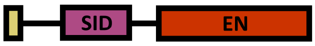	C-terminal fusion of EH1 and SID domain with repression domain Engrailed; deleted FHD [this work]
