# Supplementary material for: FoxH1 represses miR-430 during early embryonic development of zebrafish via non-canonical regulation
Source: BMC Biol. 2019 Jul 30;17:61. doi: 10.1186/s12915-019-0683-z (PMC6664792; doi:10.1186/s12915-019-0683-z)
Supplement: Supplementary file 7 — Figure S2. Regulation of CAN-target pitx2 and fgf3 by different constructs. (PDF 303 kb) [file 12915_2019_683_MOESM7_ESM.pdf]

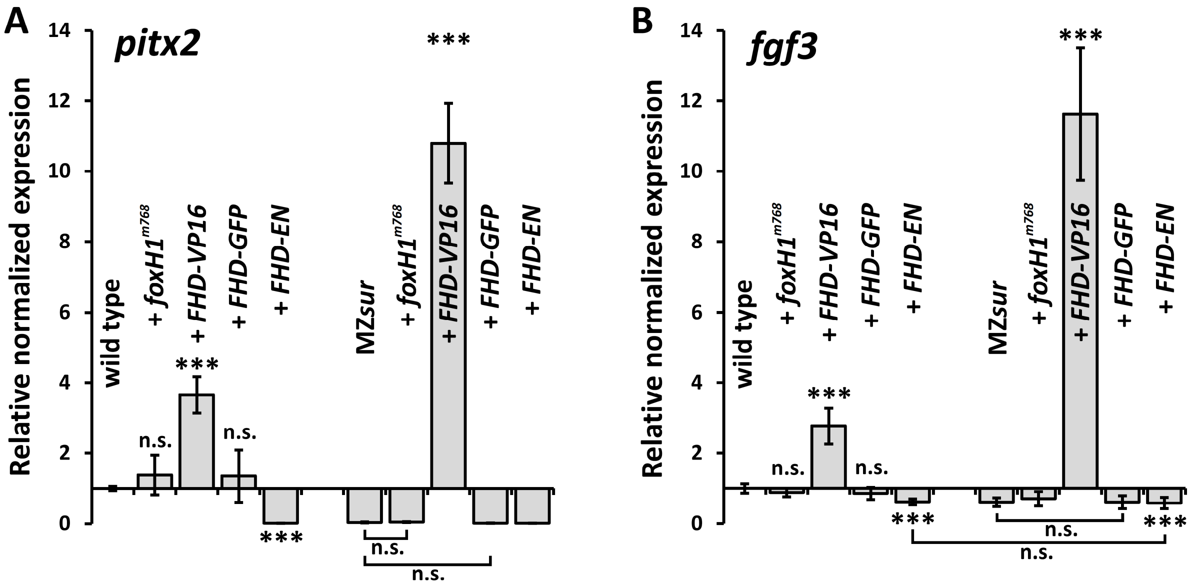

Fig. S2:

Regulation of CAN-target *pitx2* and *fgf3* by different constructs. CAN-FoxH1 targets *pitx2* (*a*) and *fgf3* (*b*) are highly upregulated following injection of *FHD-VP16* in wild type and MZ*sur* mutant embryos. Injection of *FHD-EN* leads to massive downregulation, *FHD-GFP* as well as injection of *foxH1*^{m768} shows no effect compared to uninjected control. Error bars indicate standard error (SEM) from biological triplicates. Bio-Rad CFX Manager 3.1 software was used for calculation of Relative Normalized Expression, standard error and significance (n. s.: p≥0.05; ***: p<0.001). Individual values see also Additional file 5.xlsx: Individual qPCR values.
